# Serplulimab combined with bevacizumab and chemotherapy as first-line treatment for recurrent or metastatic cervical cancer (ASTRUM-CC01): a phase II trial

**DOI:** 10.3389/fimmu.2026.1923961

**Published:** 2026-08-18

**Authors:** Hong Liu, Dengfeng Wang, Jie Tang, Chunyan Wang, Xiumin Li, Ge Lou, Guonan Zhang

**Affiliations:** 1 Department of Gynecologic Oncology, Sichuan Cancer Hospital and Institute, Chengdu, China; 2 Department of Gynecologic Oncology, Hunan Cancer Hospital, Changsha, China; 3 Department of Gynecology, Liaoning Cancer Hospital and Institute, Shenyang, China; 4 Department of Gynecology, Linyi Cancer Hospital, Linyi, China; 5 Department of Gynecology, Harbin Medical University Cancer Hospital, Harbin, China

**Keywords:** ASTRUM-CC01, combine therapy, PD-1 inhibitor, R/M cervical cancer, serplulimab

## Abstract

**Background:**

Cervical cancer is one of the leading causes of cancer-related mortality among women worldwide. While immune checkpoint inhibitors combined with bevacizumab and chemotherapy are emerging as the standard first-line therapy for recurrent or metastatic (R/M) cervical cancer, substantial unmet needs remain.

**Methods:**

ASTRUM-CC01 (NCT05444374) is a multicenter, single-arm, phase II trial. Eligible patients were aged 18–75 and had no prior systemic treatment. Patients received 4–6 cycles of serplulimab and bevacizumab combined with cisplatin and paclitaxel, followed by maintenance serplulimab and bevacizumab. The primary endpoint was objective response rate (ORR); secondary endpoints included progression-free survival (PFS), overall survival (OS), duration of response (DoR), and safety.

**Results:**

A total of 48 patients were enrolled; 43 were evaluable for efficacy. The ORR was 75.00% (95% CI, 60.40-86.36), and the disease control rate (DCR) was 89.58% (95% CI, 77.34-96.53) in the overall population. The median PFS was 21.23 months (95% CI: 14.43-NR); OS data were immature, with an estimated 12-month OS rate of 83.97% (95% CI: 73.66-95.72). Subgroup analysis indicated that high baseline clinical stage at initial diagnosis was a risk factor for PFS (HR = 3.01, *P* = 0.023); other baseline characteristics, including PD-L1 expression levels and the location of recurrence, did not significantly correlate with survival. No new safety signals were observed. Grade ≥r adverse events occurred in 21 patients (43.75%), including fistula events in 5 patients (10.42%).

**Conclusion:**

First-line treatment with serplulimab in combination with bevacizumab and chemotherapy demonstrated promising antitumor activity and acceptable safety in R/M cervical cancer. These findings support further evaluation in phase III trials.

## 1 Introduction

Cervical cancer is the most common malignancy among the three major cancers of the female reproductive system and remains the fourth leading cause of cancer-related death in women worldwide, with a particularly heavy burden in developing countries. According to recent epidemiological data, approximately 0.66 million new cases and 0.35 million deaths were reported globally in 2022, with China accounting for approximately 151,000 new cases and 56,000 deaths (1). Despite substantial advances in the definitive management of locally advanced disease over the past decades, disease recurrence still occurs in approximately 30% of patients (2), and around 6% present with metastatic disease at initial diagnosis (3). For patients with recurrent or metastatic (R/M) cervical cancer who are no longer amenable to curative local therapies, systemic treatment remains the cornerstone of management; however, clinical outcomes remain suboptimal. The historical standard regimen of platinum-based chemotherapy combined with bevacizumab yields a median overall survival (OS) of only 13–17 months (4).

In recent years, the incorporation of immune checkpoint inhibitors (ICIs) has offered a promising strategy to overcome the therapeutic limitations of chemotherapy with anti-angiogenic therapy. Anti-angiogenic therapy has been shown to normalize tumor vasculature, enhance immune cell infiltration, and convert “cold” tumors into “hot,” thereby synergizing with immunotherapy (5). The landmark phase III KEYNOTE-826 trial has established pembrolizumab combined with chemotherapy, with or without bevacizumab, as a new first-line standard of care for R/M cervical cancer. However, the use of bevacizumab was determined by the investigators (6, 7). Therefore, whether adding a PD-1 inhibitor to the current standard of chemotherapy plus bevacizumab can provide additional survival benefits still lacks consistent evidence. Furthermore, the BEATcc trial evaluated the efficacy of adding PD-L1 inhibitor atezolizumab in a setting where all patients received anti-angiogenic combined chemotherapy and demonstrated a significant survival benefit, further supporting the rationale for immunotherapy combination strategies (8). Nevertheless, it is important to note that PD-L1 and PD-1 inhibitors differ in their binding targets and biological mechanisms. Whether PD-1 inhibitors can achieve comparable or even superior efficacy within this treatment paradigm remains to be elucidated.

Serplulimab is a humanized anti–PD-1 monoclonal antibody that has demonstrated robust antitumor activity across multiple malignancies, including small cell lung cancer, non–small cell lung cancer, and esophageal squamous cell carcinoma, with a favorable safety profile (9–11). It has been approved by the National Medical Products Administration (NMPA) for several indications, including MSI-H solid tumors and lung cancers. A prior phase II study in previously treated PD-L1–positive advanced cervical cancer showed that serplulimab in combination with nab-paclitaxel produced durable clinical responses with manageable toxicity (12). We therefore conducted this study to evaluate the efficacy and safety of first-line serplulimab in combination with HLX04 (a bevacizumab biosimilar) and chemotherapy in patients with R/M cervical cancer.

## 2 Methods

### 2.1 Study design and patients

This study was a prospective, single-arm, multicenter, phase II clinical trial (ASTRUM-CC01) led by Sichuan Cancer Hospital and conducted across five academic cancer centers in China. Eligible patients were recruited from multiple participating centers. Key inclusion criteria were as follows: female patients aged 18–75 years with histologically confirmed cervical cancer; no prior systemic antitumor therapy for R/M disease and not amenable to curative local therapy; at least one measurable lesion according to the Response Evaluation Criteria in Solid Tumors (RECIST) version 1.1; an Eastern Cooperative Oncology Group (ECOG) performance status of 0–1; and adequate organ function. Patients who had previously received platinum-based chemotherapy in the neoadjuvant, adjuvant, or concurrent chemoradiotherapy setting were eligible, provided that disease recurrence occurred more than 6 months after the last treatment. Patients with recurrence within the radiation in-field were required to have an interval of at least 3 months after completion of radiotherapy. Major exclusion criteria included prior treatment with ICIs, active autoimmune diseases, uncontrolled cardiovascular conditions, active infections requiring systemic therapy, known central nervous system metastases, or other malignancies within the past 2 years.

The study was conducted in accordance with the Declaration of Helsinki and Good Clinical Practice guidelines. The study protocol and amendments were reviewed and approved by the clinical research ethics committee of Sichuan Cancer Hospital (Approval No: SCCHEC-02-2022-085), and written informed consent was obtained from all participants. The trial was registered at ClinicalTrials.gov (NCT05444374).

### 2.2 Treatment and procedures

During the combination phase, treatment was administered every 3 weeks for 4–6 cycles. Serplulimab was given intravenously at a dose of 300 mg on day 1 of each cycle, and HLX04 (a bevacizumab biosimilar) at 7.5 mg/kg on day 1. Chemotherapy consisted of paclitaxel (175 mg/m²) and cisplatin (50 mg/m²) on day 1 of each cycle. After completion of chemotherapy, patients continued to receive maintenance therapy with serplulimab plus bevacizumab until disease progression (PD), unacceptable toxicity, withdrawal of consent, or completion of a maximum treatment duration of 2 years (up to 35 cycles). Dose modification was not permitted for serplulimab or bevacizumab; however, treatment interruption was allowed in case of treatment-related toxicities. Dose adjustments to chemotherapy were permitted based on the severity of toxicity and clinical judgment.

Tumor assessments were performed using computed tomography (CT) or magnetic resonance imaging (MRI) according to RECIST version 1.1. Imaging evaluations were conducted every 6 weeks (± 7 days) at the first 48 weeks, every 9 weeks (± 7 days) from week 48 to week 96, and every 12 weeks (± 7 days) thereafter until PD or study termination. Patients who discontinued study treatment entered a follow-up period, including a safety follow-up at 30 days (± 7 days) after the last dose, an additional follow-up by telephone at 90 days, and survival follow-up every 12 weeks until death or study end.

### 2.3 Study endpoints and assessment

The primary endpoint of this study was objective response rate (ORR), defined as the proportion of patients achieving a best overall response of complete response (CR) or partial response (PR).

Secondary endpoints included progression-free survival (PFS), overall survival (OS), duration of response (DoR) and safety. PFS was defined as the time from enrollment to the first documented PD or death from any cause, whichever occurred first. OS was defined as the time from enrollment to death from any cause; patients without a reported death at the time of analysis were censored at the date of last known survival. DoR was defined for patients who achieved CR or PR as the time from the first documented response to the first documented disease progression or death, whichever occurred first. The disease control rate (DCR) was defined as the proportion of patients who achieved CR, PR, or stable disease (SD) as their best overall response. Adverse events (AEs) were graded according to the National Cancer Institute Common Terminology Criteria for Adverse Events (CTCAE), version 5.0.

### 2.4 Statistical analysis

Descriptive statistics were used to summarize baseline characteristics, efficacy, and safety data. Continuous variables were summarized using mean, standard deviation, median, and range, while categorical variables were presented as frequencies and percentages. The ORR and DCR were analyzed by estimating the response rate and its corresponding 95% confidence interval (CI) using the Clopper–Pearson method. Time-to-event endpoints, including PFS, OS, and DoR, were analyzed using the Kaplan–Meier method, with median survival times and corresponding 95% CIs estimated. Survival rates at specific time points were also calculated based on Kaplan–Meier estimates. Median follow-up time was calculated using the reverse Kaplan–Meier method. For subgroup analyses, survival differences between groups were compared using the log-rank test, and hazard ratios (HRs) with 95% confidence intervals (CIs) were estimated using Cox proportional hazards models. All statistical tests were two-sided, with a significance level of α = 0.05. Statistical analyses were performed using R software (version 4.3.2).

## 3 Results

### 3.1 Patient disposition and baseline characteristics

**Figure 1:**
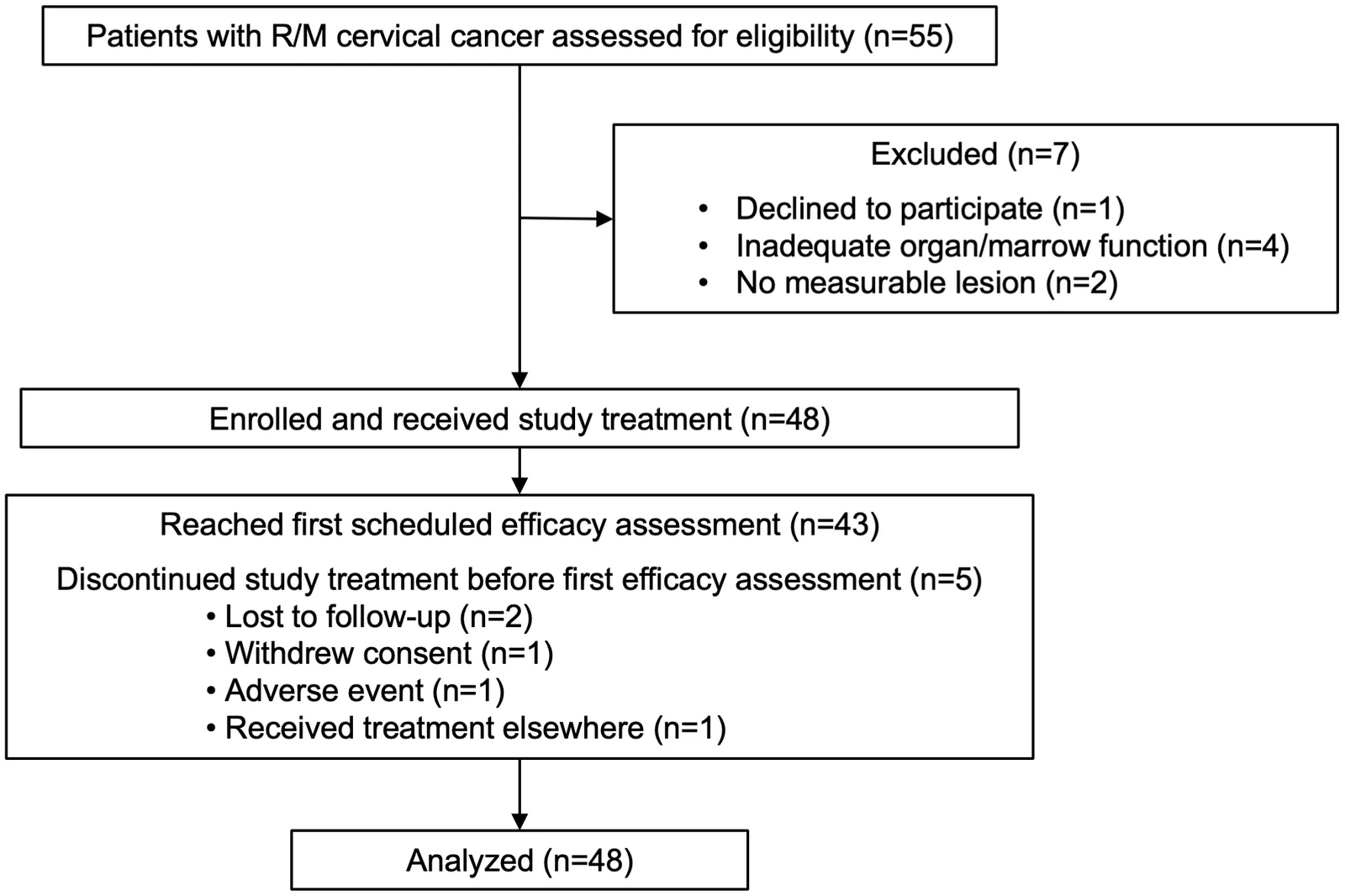
Flow diagram of patient enrollment, treatment, and analysis in the ASTRUM-CC01 trial. Of 55 patients with R/M cervical cancer assessed for eligibility, 7 were excluded before enrollment. The remaining 48 patients were enrolled and received at least one cycle of study treatment. Of these, 43 patients reached the first scheduled efficacy assessment and constituted the response-evaluable population, whereas 5 patients discontinued study treatment before the first efficacy assessment. All 48 enrolled patients who received at least one dose were included in the safety and survival analyses. R/M, recurrent or metastatic.

Between February 2023 and March 2025, after screening and exclusion, a total of 48 patients were enrolled (**Figure 1**). The baseline demographic characteristics and patients’ treatment status are summarized in **Table 1**; **Supplementary Table 1**. At study entry, the majority (42/48, 87.50%) had recurrent or progressive disease, and 6 patients (12.50%) presented with *de novo* metastatic disease; the distribution of metastatic sites included two cases (4.17%) each of lung and distant lymph node metastases, and one case (2.08%) each of pancreatic, bone, adrenal, and peritoneal involvement. Most patients were initially diagnosed at stage III (19/48, 39.58%). PD-L1 expression was available in 37 patients, among whom most patients had a combined positive score (CPS) <1 (23/48, 47.92%). Regarding prior treatments, most patients had received radiotherapy (36/48, 75.00%), and 19 patients (39.58%) had undergone prior surgery. Among patients with prior radiotherapy, out-of-field recurrence was observed in 20 patients (41.67%), while in-field recurrence occurred in 12 patients (25.00%). Pelvic and extra-pelvic recurrence were observed in 17 (35.42%) and 23 (47.92%) patients, respectively.

**Table 1:** Baseline demographics and disease characteristics.

Variable	Number (n=48)
Median age (range), years	54 (25, 68)
Median BMI (mean ± SD), kg/m²	23.01 ± 3.23
ECOG PS, n (%)	
0	25 (52.08)
1	23 (47.92)
Histological subtype, n (%)	
Squamous cell carcinoma	41 (85.42)
Adenocarcinoma	7 (14.58)
PD-L1 CPS, n (%)	
<1	23 (47.92)
≥1	14 (29.17)
Unknown	11 (22.92)
FIGO stage at initial diagnosis, n (%)	
I	10 (20.83)
II	13 (27.08)
III	19 (39.58)
IV	6 (12.50)
Prior therapy, n (%)	
Chemotherapy	37 (77.08)
Radiotherapy	36 (75.00)
Surgery	19 (39.58)
None	6 (12.50)
Pelvic recurrence, n (%)	
Yes	17 (35.42)
No	23 (47.92)
Missing	2 (4.17)
Site of recurrence after radiotherapy, n (%)	
In-field recurrence	12 (25.00)
Out-of-field recurrence	20 (41.67)
No recurrence	2 (4.17)
Missing	2 (4.17)

BMI, body mass index; ECOG PS, Eastern Cooperative Oncology Group performance status; CPS, combined positive score.

As of the data cutoff date, the study remains ongoing. The median follow-up duration was 20.13 months (range, 0.40–28.73). All patients received a median of 6 cycles (range, 1–6) of combination treatment. The median number of treatment cycles for both serplulimab and HLX04 was 10 (range, 1–35).

### 3.2 Efficacy

**Figure 2:**
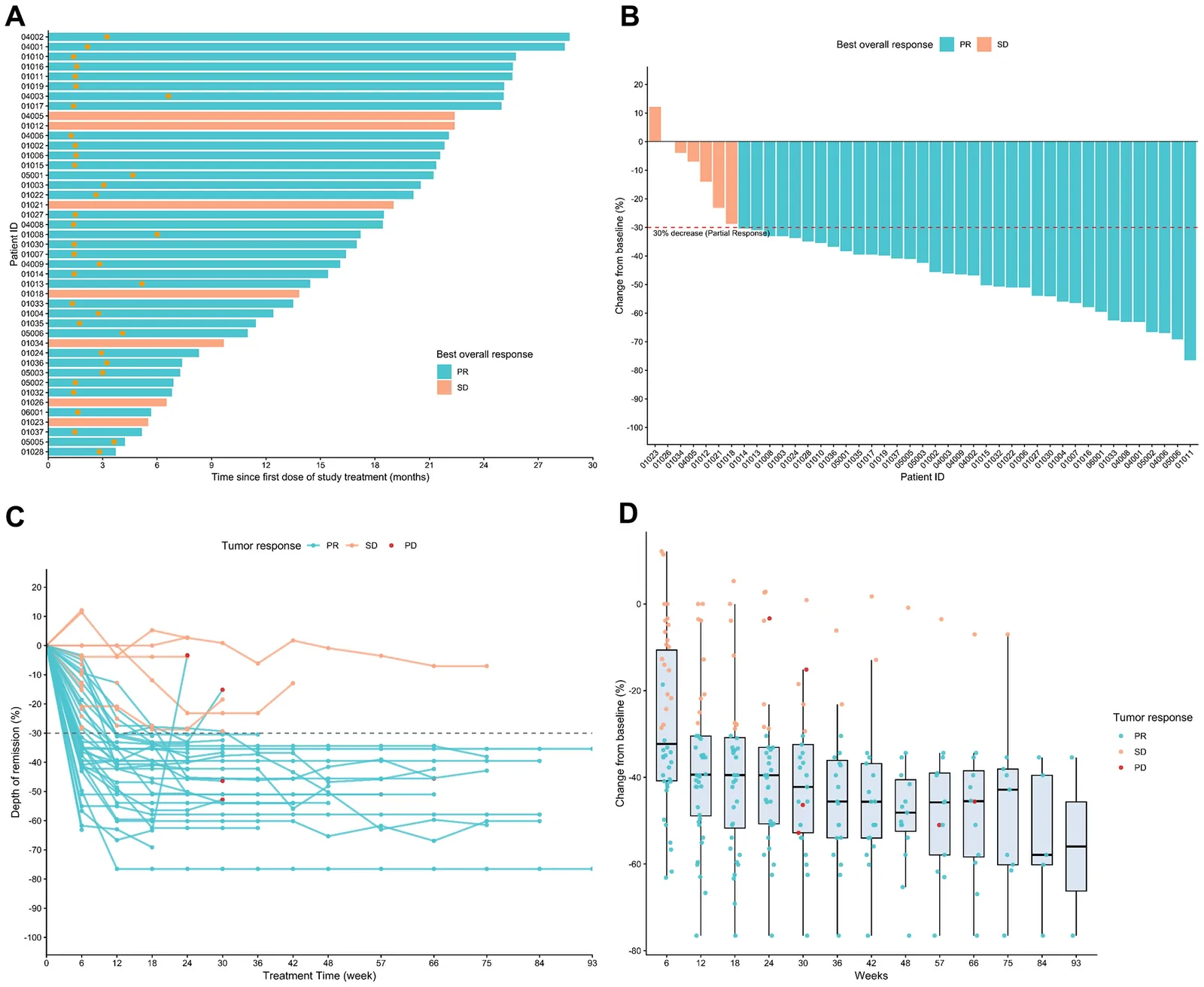
Assessment of individual tumor response and treatment durability in efficacy-evaluable patients. **(A)** Swimmer plot illustrating treatment duration and best overall response in individual patients; **(B)** Waterfall plot showing the maximum percentage change from baseline in target lesion size; **(C)** Spider plot depicting longitudinal changes in the target lesion over time; **(D)** Depth of response over time based on scheduled radiologic assessments.

At the data cutoff, among the 48 enrolled patients, 43 were evaluable for tumor response. Five patients had received only one treatment cycle and had not yet reached their first scheduled efficacy assessment. In the total population, the ORR was 75.00% (95% CI, 60.40-86.36), with 36 patients achieving PR and 7 having SD, and the DCR was 89.58% (95% CI, 77.34-96.53), as shown in **Table 2**. In the PD-L1 subgroup analysis, the ORR was 78.57% (11/14; 95% CI, 49.20%–95.34%) in patients with PD-L1 CPS ≥ 1 and 82.61% (19/23; 95% CI, 61.22%–95.05%) in patients with PD-L1 CPS < 1. Among the 43 efficacy-evaluable patients, the ORR and DCR were 83.72% (36/43; 95% CI, 69.30%–93.19%) and 100.00% (43/43; 95% CI, 91.78%–100.00%), respectively (**Supplementary Table 2**). The swimmer plot of individual patient response status and the waterfall plot of maximum percentage change in target lesion size from baseline are shown in **Figures 2A, B**. The spider plot of longitudinal changes in the sum of target lesion diameters and the depth of response over time are presented in **Figures 2C, D**.

**Table 2:** Objective responses and disease control rate in all patients.

Best response	Total (n=48)
CR	0 (0)
PR	36 (75.00)
SD	7 (14.58)
PD	0 (0)
NE	5 (10.42)
ORR (CR+PR)	36 (75.00)
95% CI	60.40-86.36
DCR (CR+PR+SD)	43 (89.58)
95% CI	77.34-96.53

CR, complete response; PR, partial response; SD, stable disease; PD, progressive disease; ORR, overall response rate; DCR, disease control rate; CI, confidence interval.

**Figure 3:**
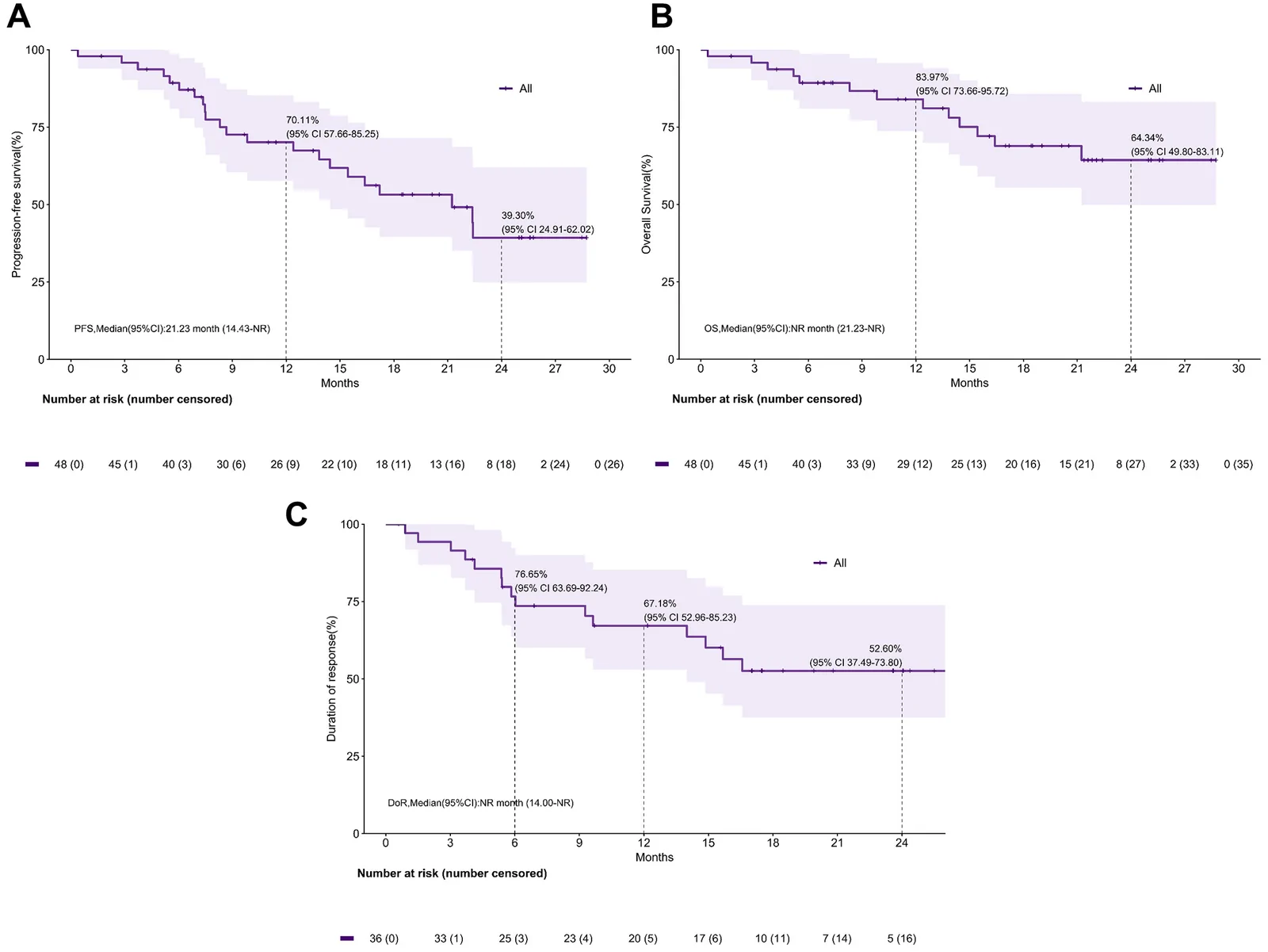
Kaplan–Meier curves of survival and duration of response. **(A)** Progression-free survival in all patients; **(B)** Overall survival in all patients; **(C)** Duration of response in patients who achieved complete or partial response.

**Figure 4:**
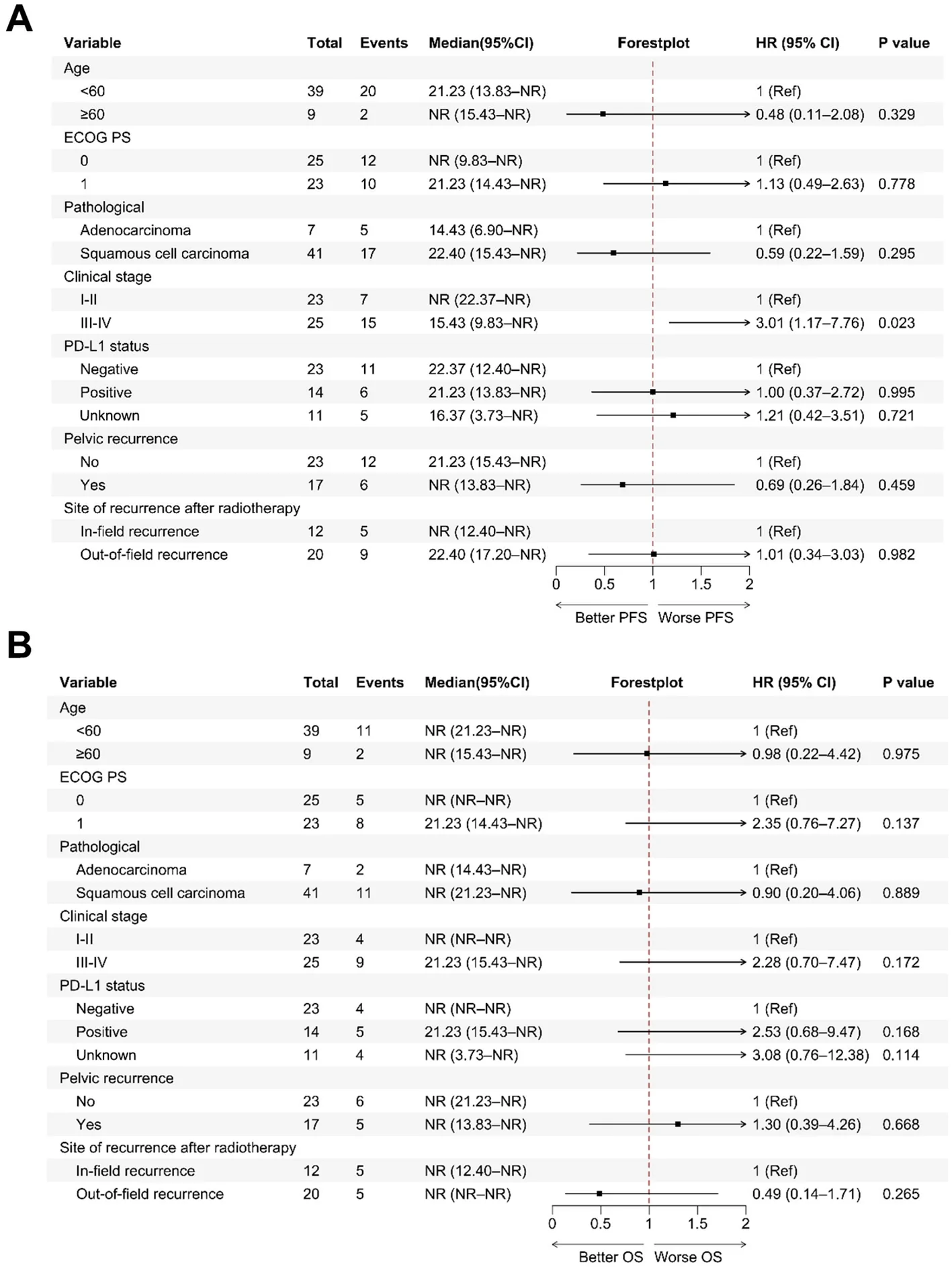
Subgroup analyses of clinical outcomes. **(A)** Forest plot of progression-free survival in all patients; **(B)** Forest plot of overall survival in all patients.

At the data cutoff, the median follow-up was 20.13 months (range, 0.40–28.73) for OS and 21.37 months (range, 0.40–28.73) for PFS. In the overall population, 22 of 48 patients (45.83%) experienced an event, with a median PFS of 21.23 months (95% CI, 14.43–not reached [NR]) (**Figure 3A**). A total of 13 OS events (27.08%) were observed; the data were not yet mature. The estimated OS rates at 12 and 24 months were 83.97% and 64.34%, respectively (**Figure 3B**). Among the 36 patients who responded, the median DoR was not reached (95% CI, 14.00–NR). The response maintenance rates were 67.18% (95% CI, 52.96%–85.23%) at 12 months and 52.60% (95% CI, 37.49%–73.80%) at 24 months (**Figure 3C**). Subgroup analyses were performed to explore factors associated with PFS and OS (**Figures 4A, B**; **Supplementary Figures 1A–H**). Baseline FIGO stage at initial diagnosis was identified as a significant factor affecting PFS (HR = 3.01, 95% CI, 1.17-7.76; *P* = 0.023). However, other baseline characteristics, including PD-L1 expression levels and the location of recurrence, did not significantly correlate with either PFS or OS in this cohort.

### 3.3 Safety

The safety profile is shown in **Table 3**. Overall, the majority of patients (91.67%) experienced grade 1–2 AEs. The most common AEs of any grade were anemia (56.25%), white blood cell decreased (45.83%), and constipation (35.42%). Grade ≥3 AEs occurred in 21 patients (43.75%), with the most common being white blood cell decreased (14.58%), neutrophil count decreased (10.42%), and lymphocyte count decreased (6.25%). Treatment-related adverse events (TRAEs) associated with the study drug (serplulimab or bevacizumab) are summarized in **Supplementary Table 3**. The safety of the combination regimen was consistent with the known safety profiles of each individual agent, and no new safety signals were identified. Serious adverse events (SAEs) are presented in **Supplementary Table 4**. The most common SAEs included anemia (8.33%), pyrexia (6.25%), and neutrophil count decreased (4.17%). AEs led to treatment discontinuation in 4 patients: 3 discontinued both serplulimab and HLX04, while 1 discontinued HLX04 alone; specific events leading to withdrawal included upper gastrointestinal hemorrhage, syncope, rectovaginal and enterovaginal fistulas, and increased enzyme levels of alanine aminotransferase (ALT), aspartate aminotransferase (AST), alanine phosphatase (ALP), and gamma-glutamyl transferase (GGT). Fistula events of grade ≥3 were observed in 5 patients (10.42%) in total; the baseline characteristics are described in **Supplementary Table 5**. Specifically, rectovaginal fistula occurred in 2 patients (4.17%), while enterovaginal fistula, rectal fistula, and vaginal fistula were each reported in 1 patient (2.08%).

**Table 3:** Any adverse events in all patients (events occurring in ≥10%).

AE	Total (n=48)	Grade 1-2	Grade ≥3
Anemia	27 (56.25)	25 (52.08)	2 (4.17)
White blood cell decreased	22 (45.83)	15 (31.25)	7 (14.58)
Constipation	17 (35.42)	16 (33.33)	1 (2.08)
Vomiting	16 (33.33)	16 (33.33)	–
Limb numbness	15 (31.25)	15 (31.25)	–
Hypertriglyceridemia	12 (25.00)	11 (22.92)	1 (2.08)
Neutrophil count decreased	10 (20.83)	5 (10.42)	5 (10.42)
Cough	10 (20.83)	10 (20.83)	–
Alopecia	10 (20.83)	10 (20.83)	–
Diarrhea	10 (20.83)	10 (20.83)	–
Urinary tract infection	9 (18.75)	8 (16.67)	1 (2.08)
Nausea	9 (18.75)	9 (18.75)	–
Platelet count decreased	9 (18.75)	9 (18.75)	–
Upper respiratory tract infection	8 (16.67)	8 (16.67)	–
Lymphocyte count decreased	8 (16.67)	5 (10.42)	3 (6.25)
Hypercholesterolemia	8 (16.67)	8 (16.67)	–
Epistaxis	8 (16.67)	8 (16.67)	–
ALP increased	7 (14.58)	7 (14.58)	–
AST increased	6 (12.50)	6 (12.50)	–
LDL-cholesterol increased	6 (12.50)	6 (12.50)	–
Hypokalemia	6 (12.50)	6 (12.50)	–
Pyrexia	6 (12.50)	6 (12.50)	–
Hypothyroidism	6 (12.50)	6 (12.50)	–
Creatinine increased	6 (12.50)	6 (12.50)	–
Anorexia	6 (12.50)	6 (12.50)	–
ALT increased	5 (10.42)	5 (10.42)	–
Hypoalbuminemia	5 (10.42)	5 (10.42)	–
Urine leukocytes positive	5 (10.42)	5 (10.42)	–
Pain	5 (10.42)	5 (10.42)	–
Pruritus	5 (10.42)	5 (10.42)	–
Sinus tachycardia	5 (10.42)	5 (10.42)	–
Hyperuricemia	5 (10.42)	5 (10.42)	–

AE, adverse event; ALP, alkaline phosphatase; AST, aspartate aminotransferase; LDL, low-density lipoprotein; ALT, alanine aminotransferase.

## 4 Discussion

Historically, the landmark GOG-240 trial established bevacizumab combined with chemotherapy as the standard first-line treatment for R/M cervical cancer, improving survival outcomes compared with chemotherapy alone (13). However, in the burgeoning era of immunotherapy, several important questions remain unresolved. In KEYNOTE-826, bevacizumab use was optional, making it difficult to isolate the contribution of the immunotherapy–anti-angiogenic combination. In contrast, BEATcc evaluated a PD-L1 inhibitor rather than PD-1 blockade. Therefore, the efficacy and safety of a PD-1 inhibitor combined with a uniform bevacizumab-plus-chemotherapy backbone remain insufficiently characterized. ASTRUM-CC01 was designed to address this clinically relevant evidence gap. Among efficacy-evaluable 43 patients, an ORR of 83.72% and a DCR of 100% were achieved, with most responses occurring as early as the first assessment at week 6, highlighting the rapid tumor shrinkage capability of this regimen. In terms of survival outcomes, the median PFS reached 21.23 months. Among responders, the median DoR was not reached, with estimated response maintenance rates of 67.18% at 12 months and 52.60% at 24 months, suggesting durable disease control. OS was also immature at the time of analysis, suggesting a potential long-term survival benefit. Notably, the treatment benefit appeared robust across clinically relevant subgroups, including recurrence pattern (in-field vs. out-of-field; pelvic vs. extra-pelvic), histological subtype, and PD-L1 expression status. Importantly, the safety profile was manageable and consistent with the known toxicities of each component, with no new safety signals observed following the addition of serplulimab. Collectively, the current results from ASTRUM-CC01 underscore the synergistic effect of combining serplulimab with anti-angiogenic therapy and chemotherapy, particularly in achieving rapid response and durable disease control, supporting its potential as a promising first-line treatment strategy for patients with R/M cervical cancer.

The clinical outcomes observed in our trial demonstrate a numerically longer PFS compared to previous landmark studies. In the total population of the phase III KEYNOTE-826 trial, the median PFS was 10.4 months (7). In contrast, our study achieved a median PFS of 21.23 months. It is plausible that the consistent use of bevacizumab across our entire study cohort played a pivotal role in this enhancement. In KEYNOTE-826, bevacizumab was administered at the investigators’ discretion, with only 63.6% of patients receiving it; notably, in the *post-hoc* analysis, in the bevacizumab-treated subgroup the median PFS improved to 15.2 months, and in the East Asian population—where bevacizumab use increased to 75.4%—the median PFS further extended to 18.0 months (14, 15). This trend aligns with our findings, suggesting a potential synergy between anti-angiogenic and ICIs in R/M cervical cancer. Mechanistically, research has shown that anti-angiogenic therapy does more than just inhibit tumor angiogenesis and cut off nutrient supply; it promotes vascular normalization, which enhances T-cell infiltration and reprograms the immunosuppressive tumor microenvironment (TME) into an immune-supportive state by reducing myeloid-derived suppressor cells (MDSCs) and overcoming dendritic cell maturation barriers (5). The BEATcc trial further validates the benefit of the PD-L1 inhibitor atezolizumab combined with bevacizumab and chemotherapy, yielding a median PFS of 13.7 months. The potential difference in treatment efficacy with our trial may stem from the distinct molecular mechanisms of the specific inhibitors used. Serplulimab is a high-affinity PD-1 monoclonal antibody (16). While both PD-1 and PD-L1 inhibitor approaches disrupt the PD-1/PD-L1 axis to restore antitumor immunity, PD-1 blockade simultaneously inhibits interactions with both PD-L1 and PD-L2, whereas PD-L1 inhibitors preserve PD-1/PD-L2 signaling, which may represent an alternative pathway for immune evasion (17). Emerging evidence from other solid tumors, including non–small cell lung cancer and gastric cancer, suggests that PD-1 inhibitors may confer deeper or more durable responses in certain contexts (18–20). It is important to acknowledge that cross-trial comparisons are limited by substantial heterogeneity and should therefore be interpreted with caution. Moreover, the PFS data are not yet mature, and extended follow-up is warranted to validate the long-term benefit. Despite these limitations, the observed improvement in efficacy outcomes provides supportive evidence of a clinically meaningful benefit of the combined strategy that incorporates serplulimab, anti-angiogenic therapy, and chemotherapy.

Patients with PD-L1–negative tumors and those with in-field recurrence are generally considered immunorefractory subgroups in R/M cervical cancer, largely due to a more immunosuppressive TME (21). Consistent with this, subgroup analyses from the KEYNOTE-826 trial demonstrated that patients with PD-L1–negative disease did not derive a significant survival benefit from pembrolizumab-based therapy (7), leading the U.S. Food and Drug Administration to restrict its first-line indication to PD-L1–positive populations. Additionally, the BEATcc trial did not report efficacy outcomes stratified by PD-L1 expression; it remains uncertain whether the survival benefit is consistent across different PD-L1 subgroups (16). In our exploratory analyses, the ORR was numerically higher in patients with PD-L1 CPS <1 than in those with CPS ≥1 (82.61% and 78.57%, respectively), and the survival benefit of this serplulimab-based regimen appeared to be consistent across PD-L1 expression subgroups. However, these findings should be interpreted with caution, given the single-arm design of this study and the limited sample size of subgroup analyses, which preclude definitive conclusions regarding whether PD-L1 status can predict differential treatment benefit. Further validation in larger, randomized studies is warranted.

The safety profile observed in this study was consistent with the known toxicities of serplulimab and bevacizumab combined with chemotherapy, with grade ≥3 AEs occurring in 43.75% of patients, predominantly manageable hematologic toxicities. Among TRAEs, fistula formation—including gastrointestinal and genitourinary fistulas—remains one of the most clinically significant and concerning complications in patients with cervical cancer receiving bevacizumab (22). The pathogenesis of fistula formation is mainly twofold: first, prior pelvic radiotherapy plays a central role by inducing tissue fibrosis, vascular damage, and chronic hypoxia, which collectively impair tissue repair capacity. Concurrently, inhibition of the vascular endothelial growth factor (VEGF) signaling pathway by anti-angiogenic therapy further compromises neovascularization and mucosal healing. In the context of rapid tumor regression following effective systemic therapy, previously infiltrated or structurally weakened tissues may become susceptible to necrosis, perforation, and subsequent fistula formation (23–25). Real-world data further support a significantly increased risk of fistula formation in cervical cancer patients receiving bevacizumab before or after radiotherapy compared to radiotherapy alone, highlighting a strong association between anti-angiogenic therapy and this complication (26). Therefore, the BEATcc trial adopted stringent eligibility criteria, excluding patients with tumor invasion into the bladder or rectal mucosa, as well as those with a history of gastrointestinal perforation or fistula within the preceding six months (16). Under such controlled conditions, fistula events of any grade occurred in 11 patients (5.4%), with grade ≥3 events reported in approximately 3%. Compared with BEATcc, ASTRUM-CC01 enrolled a less selected population, and grade ≥3 fistula events were observed in 10.42% of patients. This higher incidence may more accurately reflect the safety profile of this combination regimen in a real-world–like population, particularly among patients with prior high-dose pelvic radiotherapy, which is common in clinical practice in China. These findings underscore the importance of careful patient selection and proactive risk assessment when incorporating anti-angiogenic therapy into treatment regimens. Given these risks, prevention and early management of fistula are critical. Patients with prior extensive pelvic irradiation, full-thickness tumor invasion of adjacent organs, or pre-existing pelvic inflammation should be considered at high risk (26). During treatment, enhanced clinical surveillance is warranted, including close monitoring for early signs and symptoms such as abnormal vaginal discharge, abdominal pain, or hematuria. Once a fistula is confirmed or strongly suspected, anti-angiogenic therapy should be considered discontinued. Management should be individualized and may include nutritional support, anti-infective treatment, and multidisciplinary (MDT) surgical intervention when appropriate, to prevent progression to life-threatening complications such as peritonitis or sepsis (27).

This study has several limitations that should be acknowledged. First, the single-arm design and relatively small sample size may introduce selection bias and limit the robustness of efficacy estimates. Second, the generalizability of our findings to patients who are ineligible for anti-angiogenic therapy remains uncertain. Third, the prolonged PFS observed in this study may be associated with the consistent combination treatment strategy; however, cross-trial comparisons with prior studies should be interpreted with caution, and the magnitude of benefit cannot be definitively established in the absence of a randomized control group. In addition, OS data remain immature at the time of analysis, and a longer follow-up is required. Finally, our findings warrant further validation in a prospective, randomized controlled phase III trial, ideally with head-to-head comparisons, to better define the relative efficacy of different immunotherapy-based combination strategies.

## 5 Conclusion

In conclusion, the ASTRUM-CC01 trial demonstrated that serplulimab in combination with HLX04 (a bevacizumab biosimilar) and chemotherapy as first-line treatment exhibited robust antitumor activity and a manageable safety profile in patients with R/M cervical cancer, irrespective of PD-L1 expression status or recurrence pattern. These findings provide clinically meaningful evidence supporting the synergistic efficacy of integrating immunotherapy into anti-angiogenic therapy and chemotherapy. Nevertheless, given the single-arm design and limited sample size, further validation in large-scale, randomized phase III trials is warranted to confirm its efficacy and to better define its role in the evolving treatment for cervical cancer.

## Data Availability

The original contributions presented in the study are included in the article/**Supplementary Material**. Further inquiries can be directed to the corresponding author.
