# Supplementary material for: Serplulimab combined with bevacizumab and chemotherapy as first-line treatment for recurrent or metastatic cervical cancer (ASTRUM-CC01): a phase II trial
Source: Front Immunol. 2026 Aug 18;17:1923961. doi: 10.3389/fimmu.2026.1923961 (PMC13529689; doi:10.3389/fimmu.2026.1923961)
Supplement: Supplementary file 1 [file DataSheet1.docx]

Supplementary Material

# 1 Supplementary Figure

**Figure S1. Subgroup analyses of progression-free survival (PFS) and overall survival (OS).** PFS (A) and OS (B) according to recurrence location relative to the prior radiation field (in-field vs. out-of-field); PFS (C) and OS (D) according to anatomical site of recurrence (pelvic vs. extra-pelvic); PFS (E) and OS (F) according to histological subtype (squamous vs. non-squamous carcinoma); PFS (G) and OS (H) according to PD-L1 expression status.

# 2 Supplementary Tables

**Table S1. Treatment status**

|  | Total (n=48) |
| --- | --- |
| Combination therapy cycles, median (range) | 6 (1, 6) |
| Serplulimab treatment cycles, median (range) | 10 (1, 35) |
| HLX04 treatment cycles, median (range) | 10 (1, 35) |
| Follow-up duration, median (range), months | 20.13 months (0.40-28.73) |

**Table S2. Objective responses and disease control rate in patients with efficacy evaluation.**

|  | Total (n=43) |
| --- | --- |
| Best response |  |
| CR | 0 (0) |
| PR | 36 (83.72) |
| SD | 7 (16.28) |
| PD | 0 (0) |
| ORR (CR+PR) | 36 (83.72) |
| 95% CI | 69.30-93.19 |
| DCR (CR+PR+SD) | 43 (100) |
| 95% CI | 91.78-100 |

CR, complete response; PR, partial response; SD, stable disease; PD, progressive disease; ORR, overall response rate; DCR, disease control rate; CI, confidence interval.

**Table S3. Treatment-related adverse events** **attributed to serplulimab or bevacizumab in all patients (events occurring in ≥10%)**

| TRAE | Total (n=48) | Grade 1-2 | Grade ≥3 |
| --- | --- | --- | --- |
| Anemia | 26 (54.17) | 25 (52.08) | 1 (2.08) |
| White blood cell count decreased | 21 (43.75) | 14 (29.17) | 7 (14.58) |
| Constipation | 14 (29.17) | 14 (29.17) | - |
| Vomiting | 14 (29.17) | 14 (29.17) | - |
| Limb numbness | 13 (27.08) | 13 (27.08) | - |
| Neutrophil count decreased | 10 (20.83) | 5 (10.42) | 5 (10.42) |
| Nausea | 9 (18.75) | 9 (18.75) | - |
| Diarrhea | 9 (18.75) | 9 (18.75) | - |
| Platelet count decreased | 9 (18.75) | 9 (18.75) | - |
| Hypertriglyceridemia | 9 (18.75) | 8 (16.67) | 1 (2.08) |
| Epistaxis | 8 (16.67) | 8 (16.67) | - |
| Lymphocyte count decreased | 7 (14.58) | 4 (8.33) | 3 (6.25) |
| ALP increased | 7 (14.58) | 7 (14.58) | - |
| AST increased | 6 (12.50) | 6 (12.50) | - |
| Alopecia | 6 (12.50) | 6 (12.50) | - |
| Anorexia | 6 (12.50) | 6 (12.50) | - |
| Hypercholesterolemia | 6 (12.50) | 6 (12.50) | - |
| ALT increased | 5 (10.42) | 5 (10.42) | - |
| Hypothyroidism | 5 (10.42) | 5 (10.42) | - |
| Sinus tachycardia | 5 (10.42) | 5 (10.42) | - |
| Creatinine increased | 5 (10.42) | 5 (10.42) | - |

TRAE, treatment-related adverse event; ALP, alkaline phosphatase; ALT, alkaline aminotransferase; AST, aspartate aminotransferase.

**Table S4. Serious Adverse Events in all patients**

| SAE | Total (n=48) | Grade 1-2 | Grade ≥3 |
| --- | --- | --- | --- |
| Anemia | 4 (8.33) | 2 (4.17) | 2 (4.17) |
| Pyrexia | 3 (6.25) | 3 (6.25) | - |
| Neutrophil count decreased | 2 (4.17) | 1 (2.08) | 1 (2.08) |
| White blood cell count decreased | 2 (4.17) | - | 2 (4.17) |
| Pneumonia | 2 (4.17) | 2 (4.17) | - |
| ALT increased | 1 (2.08) | 1 (2.08) | - |
| AST increased | 1 (2.08) | 1 (2.08) | - |
| GGT increased | 1 (2.08) | 1 (2.08) | - |
| Upper gastrointestinal hemorrhage | 1 (2.08) | - | 1 (2.08) |
| Constipation | 1 (2.08) | - | 1 (2.08) |
| Vomiting | 1 (2.08) | 1 (2.08) | - |
| Enterovaginal fistula | 1 (2.08) | - | 1 (2.08) |
| Urinary tract infection | 1 (2.08) | - | 1 (2.08) |
| Cardiorespiratory arrest | 1 (2.08) | - | 1 (2.08) |
| Radiation enterocolitis | 1 (2.08) | 1 (2.08) | - |
| Syncope | 1 (2.08) | - | 1 (2.08) |
| Lymphocyte count decreased | 1 (2.08) | - | 1 (2.08) |
| Rectal hemorrhage | 1 (2.08) | - | 1 (2.08) |
| Rectal fistula | 1 (2.08) | - | 1 (2.08) |
| Rectovaginal fistula | 1 (2.08) | - | 1 (2.08) |
| ALP increased | 1 (2.08) | 1 (2.08) | - |
| Psychiatric disorders | 1 (2.08) | 1 (2.08) | - |
| Intestinal obstruction | 1 (2.08) | 1 (2.08) | - |
| Lung infection | 1 (2.08) | - | 1 (2.08) |
| Hydronephrosis | 1 (2.08) | 1 (2.08) | - |
| Gastralgia | 1 (2.08) | - | 1 (2.08) |
| Cholecystitis | 1 (2.08) | - | 1 (2.08) |
| Abdominal pain | 1 (2.08) | - | 1 (2.08) |
| Abdominal distension | 1 (2.08) | - | 1 (2.08) |
| Platelet count decreased | 1 (2.08) | 1 (2.08) | - |
| Stoma site infection | 1 (2.08) | - | 1 (2.08) |
| Vaginal hemorrhage | 1 (2.08) | - | 1 (2.08) |

SAE, serious adverse event; ALP, alkaline phosphatase; ALT, alkaline aminotransferase; AST, aspartate aminotransferase; GGT, Gamma-glutamyl transferase.

**Table S5. Baseline characteristics and clinical features of patients who developed grade ≥3 fistula events**

| Patient ID | Fistula type | Disease status | Prior pelvic radiotherapy | Recurrence pattern/site | Bladder invasion | Rectal invasion | Vaginal invasion | Time to fistula onset (months) |
| --- | --- | --- | --- | --- | --- | --- | --- | --- |
| 01018 | Rectovaginal fistula | R | Y | In-field recurrence | N | N | N | 7.43 |
| 01024 | Rectal fistula | R | Y | Out-of-field recurrence | N | N | N | 2.33 |
| 02001 | Rectovaginal fistula | R | Y | Missing | N | N | Y | 0.43 |
| 04007 | Enterovaginal fistula | R | Y | In-field recurrence | N | N | N | 0.33 |
| 06001 | Vaginal fistula | M | N | NA* | N | N | N | 2.87 |

* This patient had metastatic disease at diagnosis with no prior pelvic radiotherapy; therefore, the recurrence pattern was not applicable. R, recurrent; M, metastatic; Y, yes; N, no; NA, not applicable.
